# Biomechanics of Hollow Organs: Experimental Testing and Computational Modeling

**DOI:** 10.3390/bioengineering10020175

**Published:** 2023-01-29

**Authors:** Chiara Giulia Fontanella, Emanuele Luigi Carniel

**Affiliations:** 1 Department of Industrial Engineering, University of Padova, Via Venezia 1, 35131 Padova, Italy; 2 Centre for Mechanics of Biological Materials, University of Padova, Marzolo 9, 35131 Padova, Italy

Hollow organs are visceral organs that are hollow tubes or pouches (such as the intestine or the stomach, respectively) or that include a cavity (such as the heart) and which subserve a vital function. Hollow organs show a similar histo-anatomical organization, consisting of an epithelium surrounded by a collagen-rich connective stratum and one or multiple muscular layers [1]. These collections of tissues form structural–functional units, which are specialized to propel solids, fluids or air throughout the system. Hollow organs, such as those found in the gastrointestinal and the lower urinary tracts or the cardiovascular and respiratory systems, play fundamental roles in living organisms through their similar structures and functions. It follows that a similar approach can be adopted to investigate the functionality of different hollow organs. In this sense, bioengineering methods can provide reliable tools for analyzing functionality, considering physiological conditions, pathological and degenerative situations, and interaction phenomena with surgical and/or prosthetic devices [2,3,4,5].

The Special Issue aims to provide an overview of bioengineering activities in the field of hollow organ functionality, pathophysiology, and surgery. More in detail, the bioengineering approach frequently relies on developing models of biological structures using a coupled experimental and computational approach [6]. First, experimental activities are mandatory to investigate the histo-morphometric configuration of the biological structure. Furthermore, mechanical testing provides information relating to mechanical functionality. In vitro or ex vivo experiments are usually performed on tissue and/or organ samples from animal models, particularly swine models, human cadavers, and/or surgical scraps. Sometimes, in vivo tests are performed on animal models. Experimental activities can supply a general overview of the biological structure’s functionality. Specific experimentations can simulate surgical interventions, suggesting the response of the biological structure during and after the procedure. Furthermore, experimental activities provide the necessary data for the development, identification, and reliability assessment of computational models, such as finite element models [7]. Computational investigations, or in silico investigations, broaden experimental results and widen the possible scenarios by considering the many different conformations of the biological structure and the conditions of the biological tissues as well as many different clinical, diagnostic, and surgical activities [8]. Furthermore, computational methods find out data that experimental activities barely provide, such as the stress and the strain that the biological tissue experiences [9,10]. Such mechanical stimuli are responsible for many biomechanical processes, such as tissue damage or failure [11], tissue adaptation [12], and mechano-transduction [13]. In this sense, the bioengineering approach supplies tools that drastically reduce the economic, time, and ethical costs of the traditional medical and clinical methods of investigation.

Both experimental and computational approaches toward hollow organ functionality are reported within the Special Issue. Particular emphasis is placed on in silico methods because of their versatility and reliability in the accurate prediction of biological structure functionality, accounting for many different subject conditions and clinical and surgical situations.

Experimental activities provide the basis for the comprehension of the behavior and the functionality of a biological structure. Regarding biomechanics, both histo-morphometric and mechanical experimentations are usually performed [14,15,16]. The former are mandatory for evaluating the structural configuration of the anatomical district at both micro- and macro-structural levels. Histological analyses provide preliminary information about tissue mechanics, such as the microstructural re-arrangement phenomena that develop due to mechanical stimulation. This investigation technique allows defining the principal features of a tissue’s mechanical behavior, with particular regard to isotropic or anisotropic configuration, linear or non-linear elastic phenomena, time-dependent effects, etc. [17,18] On the other hand, a morphometric investigation by means of anatomical sections, ultrasonography, CT, and MRI data, leads to 3D CAD models of the anatomical district [19,20,21], which are mandatory for developing computational models. Mechanical tests are performed at tissue and structure levels [22,23]. Experimentations at the tissue level include tests on tissue samples accounting for different loading conditions, such as tensile, compression, shear, etc. [24,25,26]. Mechanical tests at the structure level characterize the comprehensive mechanical behavior of the specific biological structure, accounting for the specific physiological function [27,28]. Further experimental activities aim to analyze organ behavior when novel clinical and/or surgical procedures are performed [29,30].

In the case of hollow organs, general experimental activities pertain to the mechanical and structural characterization of the specific anatomical district: mechanical tests at the tissues level [31,32,33,34], such as tensile, compression, and shear tests for the investigation of nonlinear elasticity and damage phenomena; stress relaxation and creep tests for the identification of the viscoelastic behavior; mechanical tests at the sub-structural level, such as membrane indentation tests for the evaluation of in-plane bending behavior, and opening angle tests for the identification of residual stresses [35,36,37]; mechanical tests at the structural level, such as inflation tests, for the evaluation of the pressure–volume response, and flow tests for the identification of fluid-dynamic quantities and fluid–structure interaction phenomena [38,39,40,41]. Aiming to evaluate the effects of diagnostic and surgical procedures, specific experimentations can be performed. By having access to samples from animal models, human cadavers, or phantoms of the anatomical district, the structure can be sensorized to evaluate the effects of interaction with surgical and diagnostics devices and/or prostheses [42]. Furthermore, experimentations on the sample allow for analyzing the post-surgical functionality of the specific organ [43,44].

Experimental activities provide data for the development of computational models. In detail, results from mechanical tests at the tissue level, together with preliminary hypotheses from the histological analysis, determine the constitutive formulation that actually interprets tissue mechanics [17,18]. Regarding constitutive modeling, it is necessary to report the complexity of hollow organ tissue mechanics. The soft behavior entails large displacement and large strain phenomena. Thus, constitutive models must be defined in the framework of a theory capable of interpreting such geometric non-linear effects [45]. Furthermore, the micro-structural organization, which is optimized for the specific function of the tissue, entails anisotropic behavior, non-linear elastic response, and visco-elastic phenomena. Constitutive models are usually defined in the framework of anisotropic hyperelasticity and visco-hyperelasticity [37,46,47]. Data taken from mechanical tests at the tissue level are mandatory for constitutive parameter identification, which is performed using inverse analysis techniques [48]. Both the constitutive formulation of the tissues and the 3D CAD model of the anatomical district are implemented in the framework of computer-aided engineering tools, leading to the computational model. Subsequently, simulating experimental tests at the structure level makes it possible to assess the reliability of the model [6].

Computational models allow the investigation of anatomical districts in both healthy and pathologic conditions, providing information about the biomechanical functionality and the effects of pathologies [11,49,50]. Furthermore, the computational approach entails the possibility of analyzing many different diagnostic and surgical procedures, providing tools for the investigation of the interaction phenomena with devices and prostheses and for the evaluation of post-surgical functionality [8,10,49,51,52].

The papers within this Special Issue report an almost comprehensive description of the application of biomechanical methods in the framework of hollow organ functionality in health and disease. Both experimental and computational activities are reported, also considering coupled approaches.

In the paper “Variation of Passive Biomechanical Properties of the Small Intestine along Its Length: Microstructure-Based Characterization” by Sokolis (2020) [53], the author analyzes the passive function of the small intestine by means of an experimental approach. Moreover, experimentations aimed at evaluating the biomechanical properties along the length of the rat small intestine were performed. Structural experimentations were performed on tubular samples under coupling inflation and longitudinal extension loading conditions. Subsequently, an exponential fiber-reinforced hyperelastic model was implemented to interpret the experimental conditions. The minimization of the discrepancy between the model and experimental results led to constitutive parameters for the different regions of the small intestine. The paper provides an overview of the non-homogeneous mechanical properties of small intestine tissues using a coupled experimental and computational approach.

The paper “The Macro- and Micro-Mechanics of the Colon and Rectum I: Experimental Evidence” by Siri et al. (2020) [54] provides a review of the experimental characterization of large intestine biomechanics. Microscopic imaging techniques have been used to provide information about the micro-structural configuration of the tissue, with particular regard to the abundance and orientation of collagen and muscular fibers. Furthermore, experimental activities for the identification of tissue mechanical responses along both longitudinal and circumferential directions have been described. Finally, the paper summarizes the distribution of mechanical properties within the different regions of the colon and rectum.

In the paper “The Macro- and Micro-Mechanics of the Colon and Rectum II: Theoretical and Computational Methods” by Zhao et al. (2020) [55], a review of constitutive and computational modeling of the mechanics of the colon and rectum is reported. Furthermore, a specific constitutive analysis of colon and rectum tissues was performed by assuming a microstructurally based anisotropic and exponential hyperelastic formulation. Further modeling activities are reported regarding mechanotransduction, with particular regard to the stretch activation of colorectal afferent endings.

The paper “Biomechanical Force Prediction for Lengthening of Small Intestine during Distraction Enterogenesis” by Hosseini and Dunn (2020) [56] reports a coupled experimental and computational approach. More in detail, a novel distraction enterogenesis intervention was defined for the treatment of short bowel syndrome. The treatment was investigated using animal models. Contemporarily, a computational model of the anatomical site was developed, and the specific surgical intervention was simulated. The computational results were particularly interesting for the evaluation of stress and strain fields within biological tissues. Furthermore, the computational approach allowed the evaluation of the parameters of the surgical devices, such as the required distention force, depending on subject characteristics.

In the paper “Biomechanical Investigation of the Stomach Following Different Bariatric Surgery Approaches” by Toniolo et al. (2020) [57], a biomechanical approach to bariatric surgery is proposed. A computational model of the stomach was developed based on experimentations on both the swine animal model and human residual tissues. The model was exploited to analyze the influence of different bariatric techniques on stomach functionality. In detail, the computational approach made it possible to quantify the pressure–volume behavior of the stomach and the stress and strain fields within the gastric wall, which are related to the mechanisms of satiety. In this sense, computational modeling allows for the evaluation of the success of bariatric interventions.

In the paper “A Preliminary Validation of a New Surgical Procedure for the Treatment of Primary Bladder Neck Obstruction Using a Computational Modeling Approach” by Serpilli et al. (2020) [58], a novel surgical procedure for the treatment of primary bladder neck obstruction with the maintenance of anterograde ejaculation is proposed. A computational model of the anatomical district, such as the fibrotic internal urethral sphincter, was developed based on data from hysto-morphometric analysis and mechanical tests at the tissue level. Subsequently, the model was exploited to evaluate the sphincter functionality depending on different surgical parameters.

The paper “Numerical Models Can Assist Choice of an Aortic Phantom for In Vitro Testing” by Comunale et al. (2020) [59] reports computational activities that aimed to optimize phantoms for the in vitro experimentation of blood flow within the aorta. In detail, fluid–structure interaction analyses were performed to evaluate the influence of phantom wall material on fluid flow. The approach is interesting because of the exploitation of computational techniques for the optimal design of experimental devices and procedures.

In the paper “Novel Bionics Assessment of Anorectal Mechanosensory Physiology” by Gregersen (2020) [60], a novel diagnostic device is reported. Once placed within the rectum, the proposed tool measures pressure, bending, and shape changes. Such data are useful for studying defecation patterns in pathologic patients, with particular regard to subjects with fecal incontinence and constipation. The device has been designed to account for biomechanical and biomechatronical principles.

Finally, the paper “Biomechanics Assist Measurement, Modeling, Engineering Applications, and Clinical Decision Making in Medicine” by Chi et al. (2023) [61] reports an overview of engineering methods for medicine, with a particular focus on hollow organs and structures. Attention is paid not only to pre-clinical planning of interventions but also to engineering methods for a more rational clinical decision-making process.

Currently, the application of biomechanical analysis has been broadened to many different fields: the evaluation of the anatomical district functionality in health and disease, the definition of diagnostic techniques and devices, the design of surgical instrumentations and prostheses, the pre-surgical planning of interventions, etc. Regarding specific biomedical fields, biomechanical methods have found wide applications in orthopedics or cardio-vascular biomechanics. Nowadays, biomechanical interest is also focusing on other biomedical fields, with regard to the gastrointestinal region and the urinary tract.

Hollow organs include a variety of biological structures from many different anatomical regions. A similar approach can be adopted for the experimental and computational investigation of functionality in health and disease. On the other side, specific techniques must be adopted for the computational and/or experimental investigation of diagnostic and surgical procedures. The Special Issue aims to provide a general overview of the mentioned topics.
